# Perceived quality of parent-child interaction in parents of autistic children: relationship with parental education level

**DOI:** 10.3389/fpsyt.2024.1433823

**Published:** 2024-10-23

**Authors:** Feiying Wang, Wenchong Du, Lai-Sang Iao

**Affiliations:** ^1^ Child Health Section, Affiliated Maternity and Child Health Care Hospital of Nantong University, Jiangsu, China; ^2^ NTU Psychology, Nottingham Trent University, Nottingham, United Kingdom

**Keywords:** autism, parent-child interaction quality, parental education level, Chinese, non-WEIRD countries

## Abstract

**Introduction:**

Most autism research was conducted in Western, Educated, Industrialised, Rich and Democratic (WEIRD) countries. This study is the first to examine factors that were associated with perceived quality of parent-child interaction in non-WEIRD parents of autistic children.

**Methods:**

Ninety-one Chinese parents of autistic children (82 females, 9 males) completed an online survey which involved two sections. The first section included demographics questions about the parent and the family, including age, gender, educational level, and household income. It also assessed parents’ perceived quality of parent-child interaction and their autistic traits. The second section included demographics questions about their autistic child, including age and gender, and assessed autistic traits and behavioural problems.

**Results:**

Parent’s education level was associated with and the only predictor of their perceived quality of parent-child interaction. The higher educational level in parents the higher quality of parent-child interaction was perceived by the parents.

**Discussion:**

These findings underscored the significance of parents’ education level as a unique predictor of perceived parent-child interaction quality among Chinese parents of autistic children, providing implications to corresponding education and social policies in China and other non-WEIRD countries.

## Introduction

Autism is clinically characterised by difficulties in social interaction and communication, alongside repetitive behaviours and restricted interests (1). Children diagnosed with autism may also exhibit additional behavioural problems such as hyperactivity and anxiety (e.g., 2, 3), which have been shown to negatively impact the quality of parent-child interaction (e.g., 4, 5). Research indicates that these interactions are not only pivotal for the child’s development (e.g., 6, 7) but also significantly affects parents’ mental health (e.g., 8, 9). Understanding the factors that contribute to the quality of this interaction in families with autistic children is of global importance.

Lower-quality interaction with parents was found in autistic children compared to typically developing children and children with other developmental conditions (10, 11). From as young as 10 months, infants at elevated likelihood for autism showed fewer initiations of interaction with their parents (12), and by 11 months, those later diagnosed with autism showed decreased social engagement in free play with their parents (13). Non-social autistic traits exhibited by infants at elevated likelihood for autism who were later diagnosed with autism, such as attention disengagement, may also negatively influence the quality of parent-child interaction (14, 15). Additionally, it was found that the level of autistic traits and behavioural problems demonstrated in autistic children aged 2 to 12 years was correlated with lower quality of parent-child interaction (4, 5, 16). However, it is critical to acknowledge that parent-child interaction is a reciprocal process that involves the responsive behaviours of both the child and parent within the context of their social ecology (17). Consequently, the quality of this interaction in families with autistic children cannot be attributed solely to the child characteristics including autistic traits and behavioural problems. A comprehensive assessment must also account for parent characteristics and the family’s circumstances, all of which operate within and are shaped by a broader ecological framework. This ecological framework includes attitudes and ideologies of a culture (6).

In infants with an increased likelihood of an autism diagnosis, their parents have been observed to demonstrate greater directiveness and less sensitivity in their responses, potentially influencing the dynamics of early interactions (18, 19). Parental interaction styles, including increased directiveness, have been variably beneficial, particularly in promoting higher engagement and participation in play among children with certain developmental challenges (20). These styles reflect adaptations to the child’s diagnosis, age, and language level, suggesting a nuanced landscape of parenting behaviours that are responsive to the unique developmental trajectories of autistic children. Furthermore, parents of autistic children may encounter their own social-communicative obstacles and display subclinical autistic traits (21, 22). Such subclinical autistic traits, often referred to as Broad Autism Phenotype (BAP), which includes aloofness, rigidity, and pragmatic language difficulties (23), can complicate the caregiving experience. For instance, mothers of autistic children with higher BAP scores reported more parenting challenges (24). This finding may be explained by the relationships between specific BAP traits and certain parenting practices; for instance, pragmatic language impairments can hinder effective communication with the child, aloofness has been associated with a reduction in positive behaviours like praise, and high levels of rigidity can lead to a tendency to dismiss the child’s emotions (25). However, Parr et al. (26) reported no significant difference in mother-child interaction quality based on the presence of BAP traits, though mothers with more pronounced BAP traits exhibited less enhancement in interaction quality following a parent-mediated intervention. As such, the relationship between BAP traits and perceived quality of parent-child interaction in parents of autistic children remains uncertain.

Parent-child interaction quality is also known to be influenced by another parent characteristics namely educational attainment and a family factor namely household income in the context of both typical development and developmental disabilities (e.g., 27–29). For instance, De Falco et al. (28) showed that mothers with higher education level were more likely to be able to provide a supportive framework for parent-child interaction while appropriately scaffolding child play. However, the associations between parent-child interaction quality, parental education level and household income have rarely been examined in the context of autism. To our knowledge, there has only been one study that reported no associations between such variables in a small sample of autistic children and their parents (30). A larger sample of autistic children and their parents may be needed to clarify whether parental education level and household income were associated with parent-child interaction quality. There is also a need to consider the ecological framework that may have shaped the research findings.

Most autism research was conducted in Western, Educated, Industrialised, Rich and Democratic (WEIRD) countries. People in these countries typically acknowledge the neurological underpinnings of autism (e.g., 31, 32) whereas people in non-WEIRD countries attribute autism to inadequate parenting practices (33, 34) or preternatural reasons such as being possessed by evil spirits (35, 36). Their beliefs on the nature of autism and the stigma associated with it affect their judgments of an autistic child, parental behaviour and other aspects involving the child and the parent, e.g., parent-child interaction and family stress (37). In non-WEIRD countries such as China where people attribute autism to inadequate parenting practices (33, 34), parents of autistic children may perceive themselves playing a significant role in their interaction with their autistic child. They face significant pressure to alter their interactions with their children, as documented by Chen (38). Moreover, Chinese parents often adopt the role of their child’s educator, and their effectiveness in this role, as well as their understanding of autism, may be influenced by their education level (39). The limited availability and high costs of special educational and social services in China compound the challenges faced by these parents, especially those with lower household income who may need to quit their jobs to care for and educate their autistic child (40–42). High family financial burdens could impede parents’ interaction with their autistic children. Consequently, factors such as the parent’s education and household income may influence the perceived quality of interaction with autistic children in Chinese families, contrasting with Ruble et al.’s findings (30). Without an examination of these possible relationships in Chinese populations, Chinese practitioners may adopt the research findings obtained from WEIRD countries and assume that addressing child characteristics may support perceived quality of parent-child interaction in Chinese parents of autistic children. Despite the high cost of services, their effectiveness on supporting perceived quality of parent-child interaction in Chinese parents of autistic children could be low. There is thus a need to investigate the factors that contribute to the perceived quality of parent-child interaction in Chinese parents with autistic children.

This study sought to identify the unique and combined factors associated with the perceived quality of parent-child interaction among Chinese parents of autistic children. While previous literature suggests links between this perceived quality and child-specific traits such as autism and behavioural problems (4, 5, 16), the associations with parents’ autistic traits and education level as well as household income remain unclear. As the first study accounting for child, parent and family characteristics simultaneously in understanding perceived quality of parent-child interaction in parents of autistic children, this study was exploratory. The insights gained from the current study aim to inform the development of targeted interventions and public policies to improve parent-child interaction in autism.

## Methods

### Participants

Ninety-three Chinese parents of autistic child aged 5 to 15 years were recruited for an online survey from Rudong Hanling Children’s Rehabilitation Center and Qidong Qianfan Children’s Rehabilitation Center in Nantong, China. Each participant confirmed their status as the main caregiver of at least one autistic child and provided informed consent before commencing the survey. Of these participants, 2 were excluded due to incomplete responses, resulting in a final sample of 91 parents (82 females, 9 males; see **Table 1** for detailed sample characteristics). Participants did not receive incentive for their participation in the survey. Ethical approval was obtained from the university ethics committee before recruitment commencing.

**Table 1:** Mean, standard deviation and range of all variables.

	Mean/%	SD	Range
Parent			
Age	37.04	5.73	26-53
Education level			
Junior high school	41.76%		
Senior high school	28.57%		
Graduate	26.37%		
Postgraduate	3.30%		
Household Income			
Less than 30,000 RMB	18.68%		
30,000 – 60,000 RMB	49.45%		
60,000 – 150,000 RMB	13.19%		
More than 150,000 RMB	18.68%		
Quality of parent-child interaction	3.07	0.63	1-5
Autistic traits (BAPQ)	3.17	0.60	1.5-5.22
Child			
Age	8.96	2.41	5-15
Gender			
Male	80.22%		
Female	19.78%		
Autistic traits (AQ-10)	6.15	1.91	1-10
Behavioural problems (CBCL)	67.22	33.90	0-203

BAPQ, Broad Autism Phenotype Questionnaire (Clinically significant value = 3.15); AQ-10, Autism-Spectrum Quotient-10 (Clinically significant value = 6); CBCL, Child Behavior Checklist.

### Measures and procedure

The online survey was cross-sectional and involved two sections. The first section included demographics questions about the parents and the families, including age, gender, education level, and household income. It also assessed parents’ perceived quality of parent-child interaction using one subscale of the Parenting Stress Index-Short Form (PSI-SF; 43, 44) and explored parents’ autistic traits using the Broad Autism Phenotype Questionnaire (BAPQ; 45). The second section collected demographics information about their autistic child, including age and gender, and evaluated autistic traits using the short Autism-Spectrum Quotient (AQ-10; 46) and behavioural problems using the Child Behaviour Checklist (higher score indicated more problems; 47). The completion of the survey was estimated to take around 20 minutes.

The Parenting Stress Index-Short Form (PSI-SF) is frequently used for assessing parental stress in those caring for autistic and non-autistic children (e.g., 44, 48). This questionnaire includes 36 items divided into three subscales: Parental Distress (PD), Parent-Child Dysfunctional Interaction (PCDI), and Difficult Child (DC). A modified 15-item version, developed specifically for Chinese parents, demonstrated strong psychometric properties and was highly correlated with the original PSI-SF (44). The PCDI subscale of this version was therefore used in this study to assesses the perceived quality of Chinese parent-child interaction. The subscale consists of 5 items using a 5-point Likert scale (5= strongly agree to 1= strongly disagree), and a higher mean score of the 5 items indicates lower quality. Sample items included “Most times I feel that my child likes me and wants to be close to me” and “My child rarely does things for me that make me feel good”. The Chinese version of the PSI-SF has good internal consistency (*α* ≥ 0.80) and concurrent validity with the Multidimensional Social Support Scale (*r* = -0.34; 49).

The BAPQ is used to evaluate subclinical traits of autism, especially in parents of autistic children (50, 51). This questionnaire is composed of three subscales, namely aloof, rigidity, and pragmatic language with a total of 36 items. Participants are required to rate each item on a 6-point Likert scale (1= very rarely to 6= very often). The sample items include “I like being around other people”, “I am comfortable with unexpected changes in plan” and “People have to talk me into trying something new”. A higher mean score of the 36 items on the questionnaire indicates higher level of autistic traits. The Chinese version of the BAPQ showed good reliability and validity (*α* = 0.78; 52).

The Chinese translation of the short ten-item version of the Autism Spectrum Quotient for children (46) is a parent-report questionnaire that assesses autistic traits in children. It consists of 10 descriptive statements about preferences and habits. The sample items in the questionnaire include “S/he usually concentrates more on the whole picture, rather than the small details” and “S/he finds it easy to go back and forth between different activities”. For each item, participants select one of four response options: “definitely agree,” “slightly agree,” “slightly disagree,” or “definitely disagree”. However, the four options are grouped and scored as either 0 or 1. A higher total score of the 10 items indicates the presence of more autistic traits. The Chinese version of the AQ-10 for children demonstrated satisfactory internal consistency (*α* = 0.79–0.84) and concurrent validity with the Social Responsiveness Scale (*r* = 0.79–0.81; 53).

The Child Behaviour Checklist (CBCL) is a widely used parent-completed assessment for rating behavioural, emotional, and social problems in children and adolescents (47). The CBCL consists of 113 questions. Each question is scored using a 3-point Likert scale (0 = absent, 1= occurs sometimes and 2= occurs often). It provides summed scores for three summary scales that include: internalising, externalising, and total problems (maximum achievable score = 226). It is widely used for clinical assessment in China and its reliability in the Chinese population was found to be 0.76 for internalising, 0.81 for externalising, and 0.83 for total problems (54–56). Multiple studies have also confirmed its validity (57, 58).

## Results

**Figure 1:**
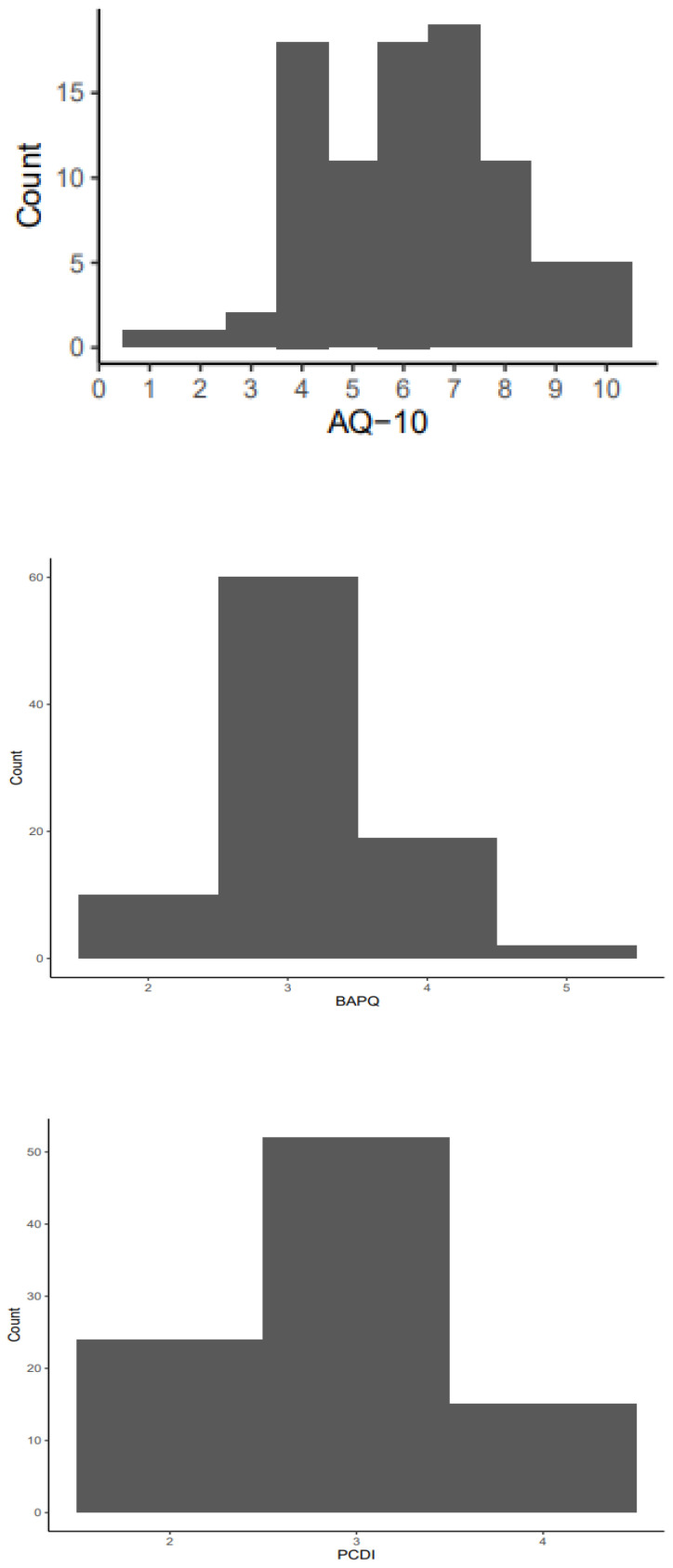
Distribution of the scores of the Autism-Spectrum Quotient-10 (AQ-10), the Broad Autism Phenotype Questionnaire (BAPQ) and the Parent-Child Dysfunctional Interaction (PCDI).

Descriptive statistics were calculated and presented in **Table 1**. The mean AQ-10 score and the mean BAPQ score were slightly above the cut-off of 6 and 3.15 suggested in Allison et al. (46) and Hurley et al. (45) respectively. **Figure 1** shows the distribution of the AQ-10, BAPQ and PCDI scores. The majority of the parents did not have a graduate degree and had relatively low household income. Parents’ ratings on perceived quality of parent-child interaction and child behavioral problems were comparable to previous studies (e.g., 48, 59).

Correlations of key measures are presented in **Table 2**. It was found that higher parent education level was associated with lower PCDI score, lower BAPQ score, higher household income, and higher AQ-10 score. Lower BAPQ was also associated with lower CBCL score. Multiple regression analysis was then conducted to investigate the extent to which the PCDI score could be predicted by key measures and other demographics (i.e., age and gender of both parent and child) given their potential influence on parent-child interaction (e.g., adolescents tend to spend less time with parents than younger children; 60).

**Table 2:** Pearson correlations among key measures.

	1	2	3	4	5	6
1. PCDI	—	.14	-.25*	.03	.05	.20
2. BAPQ		—	-.23^*^	-.04	.01	.22^*^
3. Education level			—	.32^**^	.28^**^	-.12
4. Household income				—	-.12	.04
5. AQ-10					—	-.08
6. CBCL						—

PCDI, Parent-Child Dysfunctional Interaction; BAPQ, Broad Autism Phenotype Questionnaire; AQ-10, Autism-Spectrum Quotient-10; CBCL, Child Behavior Checklist.

*p <.05; **p <.01.

There was independence of observations as indicated by a Durbin-Watson statistic of 2 (61). VIF was less than 10 and Tolerance was greater than .1 for all variables indicating an absence of multicollinearity (62). The Breush-Pagan test revealed no evidence of heteroscedasticity. Residuals were normally distributed based on a Normal Q-Q plot, A total of 10% of variance of the PCDI score was explained by the regression model (Multiple *R* ^2^ = .19, Adjusted *R* ^2^ = .10; see **Table 3**). The overall association between the predictors and the PCDI score was significant, *F*(9, 81) = 2.12, *p* <.05. **Table 3** showed that the PCDI score was uniquely predicted by parent’s education level while all other predictors were controlled for.

**Table 3:** Multiple regression analysis of the contribution of all predictors to parent-child dysfunctional interaction.

	Parent-Child Dysfunctional Interaction			
	Estimate	Std. error	*t*	*p*
Parent				
Age	-0.01	0.01	-0.43	0.67
Gender	-0.46	0.23	-2.03	0.05
Education level	-0.22	0.08	-2.57	0.01*
Household income	0.12	0.07	1.65	0.10
Autistic traits	-0.03	0.11	-0.25	0.80
Child				
Age	0.03	0.03	0.95	0.34
Gender	0.19	0.16	1.19	0.24
Autistic traits	0.06	0.04	1.67	0.10
Behavioural problems	0.002	0.002	0.88	0.38

Gender was coded 1 for females and 2 for males.

*p < .05.

## Discussion

This study is the first to identify factors that were associated with perceived quality of parent-child interaction in Chinese parents of autistic children. Results of the bivariate correlation analysis and the multiple regression analyses were consistent, which indicated that parent’s education level was associated with and the only significant predictor of their perceived quality of parent-child interaction. Higher education levels in parents were significantly linked to improved interaction quality, as evidenced by lower scores on the Parent-Child Dysfunctional Interaction scale. This insight provides implications to intervention strategies for Chinese parents of autistic children and public policies on improving overall education levels in China and other non-WEIRD countries.

Our findings are consistent with those reported in the literature of typical development and developmental disability that parent’s education level was positively associated with parent-child interaction quality (e.g., 27, 63). However, our findings diverge from those of Ruble et al.’s (30), who found no association between parent demographics and parent-child interaction quality in a limited sample of autistic children and parents. This inconsistency may be attributed to sample differences between the two studies. With a larger and more diverse sample, our study was better positioned to detect a significant relationship between perceived parent-child interaction quality and parental education level. Parents’ gender also showed a potential of predicting their perceived quality of parent-child interaction (*p* = .05). However, this finding needs to be interpreted with caution given that the sample was largely female (90%). As such, our results demonstrated that among Chinese parents of autistic children, perceived parent-child interaction quality was solely linked to parental education level, rather than any child-specific characteristics such as autistic traits or behavioural problems, despite the inherent bidirectional nature of parent-child interactions. This absence of significant associations between perceived quality of parent-child interaction and child autistic traits and behavioural problems contradicts prior literature (e.g., 4, 16). However, our findings reveal a positive association between parental education level and child autistic traits, suggesting that parents with higher education levels were more likely to report a greater prevalence of autistic traits in their autistic child. Meanwhile, these parents also reported higher perceived quality of parent-child interaction. These findings suggested that Chinese parents with higher education levels may possess enhanced competencies in interacting with their autistic children while acknowledging their child’s autistic traits. Previous research has similarly indicated that mothers with higher education levels in WEIRD countries tended to engage in a more mutually affective interactions with their children (27) and demonstrated better awareness of their children’s autistic traits and obtained autism diagnoses for their children (e.g., 64, 65). Therefore, it is plausible that parental education level has positive effects on autism awareness and perceived quality of parent-child interaction among WEIRD and non-WEIRD parents.

Another explanation could relate to the Chinese culture norms and perceptions regarding autism. As noted previously, Chinese parents often perceive themselves as primary educators of their children, with their level of education potentially affecting their ability to fulfil this role. This is particularly significant given the scarcity and expense of services available for autistic children in China (e.g., 40, 42). Meanwhile, prevalent societal beliefs in China attribute autism to inadequate parenting practices (33, 34) - a stigma that may be internalised by parents irrespective of their educational background. For example, participants in Su et al. (34) expressed self-doubt, with one suggesting, “I suspected it might be due to my way of teaching,” while another despite being well-educated, indicated, “You need to look to parents if the child is not taught well.” As such, Chinese parents may perceive themselves, rather than their autistic children, as primarily responsible for the quality of parent-child interaction. This perspective could explain the observed relationship between perceived parent-child interaction quality and parent’s education level in our study, wherein child characteristics did not demonstrate any significant associations. In contrast, WEIRD parents typically acknowledge the neurological underpinnings of autism (e.g., 31, 32), potentially leading them to attribute perceived interaction quality to child characteristics such as child autistic traits and behavioural problems, as reported in studies like Hobson et al. (4), rather than parent demographics as suggested in Ruble et al. (30). Nevertheless, further cross-cultural investigations are necessary to confirm such differences between WEIRD and non-WEIRD contexts.

The current study also found that perceived quality of parent-child interaction was not associated with household income despite that parents with higher education levels tended to report higher household income. Given that household income is the total income of all household members, it may not have a significant contribution to the main caregivers’ perceived parent-child interaction quality as the main caregivers’ education level may have. On the other hand, higher household income may not guarantee access to the limited and costly services available in China, particularly in the specific city from which our sample was recruited. As this study represents the inaugural exploration of factors associated with perceived quality of parent-child interaction in Chinese parents of autistic children, further investigations across diverse regions of China are needed. However, the absence of association between perceived parent-child interaction quality and household income observed in this study is consistent with Ruble et al.’s findings (30) obtained from a WEIRD sample. No association was also observed between perceived quality of parent-child interaction and parent’s autistic traits in the current study. This is consistent with Parr et al.’s (26) initial finding that there were no significant differences in mother-child interaction scores between mothers with high versus low BAP traits. Although parent’s autistic traits may associate with parenting practices and challenges (24, 25), they may not have an influence on perceived quality of parent-child interaction in both WEIRD and non-WEIRD parents of autistic children.

Nevertheless, there are other parental factors that may impact on parent-child interaction but were not considered in the current study. For example, mothers with panic disorder/agoraphobia were less sensitive during mother-child interaction than mothers without current mental disorders (66). There may also be a cross-partner effects of parental mental health on parent-child interaction. For example, mother’s parenting stress level was negatively associated with father’s warmth toward their autistic child 12 months later (67). As such, parental mental health could be a contributing factor to parents’ perceived quality of parent-child interaction, particularly in parents of autistic children who are vulnerable to negative psychological outcomes (e.g., 68, 69). Furthermore, Yu et al. (70) suggested that Chinese citizens were more likely to hold stigma beliefs towards autism compared to United States citizens. In this sense, Chinese parents of autistic children may frequently encounter stigmatised experiences, potentially impacting on their mental health and perceived quality of parent-child interaction. These links among affiliate stigma, parental mental health, and perceived quality of parent-child interaction may or may not be specific to the Chinese social context. Further research would be required to test for this possibility.

To conclude, our findings underscored the significance of parental education level as a unique predictor of perceived parent-child interaction quality among Chinese parents of autistic children. This association may be explained by the possibility that parents with higher education levels may possess enhanced competencies in interacting with their autistic children. It may also be explained by parents’ role as educators within the Chinese cultural context, coupled with prevailing societal attitudes towards autism. While there is a growing trend towards improved education levels and awareness of autism in China, disparities persist in access to essential information, resources, and services, particularly in certain regions, which can impact the perceived quality of parent-child interaction. Future studies should aim to test how these findings may be generalised across China and other non-WEIRD countries. It is also important to note that this study focused on parents’ perceived quality of parent-child interaction rather than objective measures of parent-child interaction quality. Future studies could therefore incorporate detailed observational measures to rigorously assess the quality of interactions between parent and child as well as among family members. It may also be essential to measure these interactions under a neurodivergent rather than neurotypical lens given the double empathy hypothesis, which suggests that neurotypical people have just as much difficulty in understanding neurodivergent people as vice versa (71). Future studies could also adopt longitudinal designs to elucidate the causal dynamics between key variables and parent-child interaction quality. Nevertheless, our findings provide implications to intervention strategies for Chinese parents of autistic children, focusing on developing their competencies in interacting with their autistic children despite their education level rather than addressing their child’s autistic traits and behavioural characteristics. Our findings also provide implications to corresponding education and social policies with a particular focus on improving overall education level and promoting public understanding of autism in China and other non-WEIRD countries.

## Data Availability

The raw data supporting the conclusions of this article will be made available by the authors, without undue reservation.
